# Rare diseases in Chile: challenges and recommendations in universal health coverage context

**DOI:** 10.1186/s13023-019-1261-8

**Published:** 2019-12-11

**Authors:** Gonzalo Encina, Carla Castillo-Laborde, Juan A. Lecaros, Karen Dubois-Camacho, Juan F. Calderón, Ximena Aguilera, Andrés D. Klein, Gabriela M. Repetto

**Affiliations:** 1 Centro de Genética y Genómica, Instituto de Ciencias e Innovación en Medicina, Facultad de Medicina, Clínica Alemana Universidad del Desarrollo, Santiago, Chile; 2 Centro de Epidemiología y Políticas de Salud, Facultad de Medicina, Clínica Alemana Universidad del Desarrollo, Santiago, Chile; 3 Observatorio de Bioética y Derecho, Instituto de Ciencias e Innovación en Medicina, Facultad de Medicina, Clínica Alemana Universidad del Desarrollo, Santiago, Chile; 4 Federación Chilena de Enfermedades Raras, Santiago, Chile; 5 Laboratorio de Inmunidad Innata, Programa de Inmunología, Facultad de Medicina, Instituto de Ciencias Biomédicas, Universidad de Chile, Santiago, Chile

**Keywords:** Chile, Health policy, Rare diseases, Universal health coverage

## Abstract

Rare diseases (RDs) are a large number of diverse conditions with low individual prevalence, but collectively may affect up to 3.5–5.9% of the population. They have psychosocial and economic impact on patients and societies, and are a significant problem for healthcare systems, especially for countries with limited resources. In Chile, financial protection exists for 20 known RDs through different programs that cover diagnosis and treatments. Although beneficial for a number of conditions, most RD patients are left without a proper legal structure that guarantees a financial coverage, and in a vulnerable situation. In this review, we present and analyze the main challenges of the Chilean healthcare system and legislation on RDs, and other ambits of the RD ecosystem, including patient advocacy groups and research. Finally, we propose a set of policy recommendations that includes creating a patient registry, eliciting social preferences on health and financial coverage, improving access to clinical genetic services and therapies, promoting research on RDs and establishing a Latin-American cooperation network, all aimed at promoting equitable quality healthcare access for people living with RDs.

## Background

**Fig. 1:**
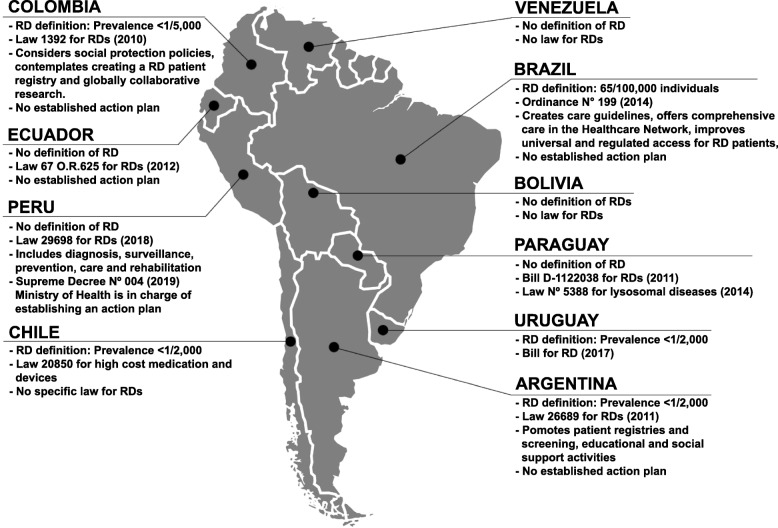
South American landscape on RD definitions and laws

A rare disease (RD) is a medical condition with low prevalence, affecting less than 1/2000 individuals according to the European Union (EU) [1]. Unlike the EU, that established a single prevalence for all its member states, in Latin America there is no consensus definition of RDs and each country uses a definition through its own national regulations or public policies. For instance, Mexico and Argentina use the EU definition, in Brazil, a rare disease is defined as one affecting fewer than 65 out of 100,000 individuals, and Peru, Chile and other countries have no clear definition of RDs [2–8]. A summary of individual South American country’s definition of RD and laws can be found in Fig. 1. Approximately 7000 RDs are recognized and 80% of the cases have an underlying genetic cause [1]. In general, RDs are severe chronically debilitating conditions that have a significant psychosocial and economic impact on patients and their families [9]. Although few people suffer from each condition, jointly they may affect 3.5–5.9% of the population [10], transforming RDs into a public health problem and a significant challenge from the right-to-health perspective and for healthcare systems [11].

The fundamental ethical question regarding RDs is how societies organize themselves to correct the inequalities that RD patients suffer derived from the “natural lottery”. Even though these disorders individually affect a small number of complex patients, the right to health of RD patients finds an obvious difficulty in the constant scarcity of resources in face of other health demands of the population.

This article presents an overview of the Chilean Health System and how it currently addresses access to diagnosis and treatment for patients with RDs, as well as other dimensions such as regulation, patients` participation and research that are relevant to the RD ecosystem. Finally, this article proposes policy recommendations to address the main challenges for the benefit of patients with RDs, supported by ethical considerations.

## The current ecosystem for RDs in Chile

### The Chilean National Healthcare System

In Chile, a country with 17,5 million inhabitants [12], the overall prevalence of RDs is unknown. Epidemiological information is primarily based on the National Health Survey that does not collect information on RDs. There is data showing that congenital anomalies are the second cause of infant mortality, with many of them presenting in the context of RDs [13].

The Chilean Healthcare System is composed of two sectors, public and private, covering about 95% of the population [14]. The government-run National Health Fund (FONASA) is funded by salary-based mandatory contributions (7%) and taxes. It provides healthcare for approximately 78% of the population, including the rural and urban poor, the lower middle class and the retirees, mainly through the National System of Healthcare Services. The private sector (twelve insurers called ISAPREs) is financed by mandatory and voluntary contributions (on average 10% of salary). ISAPREs cover approximately 14% of the population belonging to the higher income social groups and provide services mainly through private facilities. The remainder of the population is either covered under other plans (e.g., Armed Forces) or has no insurance [15].

Healthcare reform in 2005 introduced a Universal System with Explicit Guarantees in Health (GES). This system assures timely access to quality healthcare services and financial protection for a prioritized set of 80 programs, diseases or health conditions based on prevalence, severity, cost, and impact on quality of life [16]. These include pediatric cancers, breast cancer, chronic heart disorders, HIV/AIDS and diabetes among others. GES also covers treatment for two RDs: cystic fibrosis and hemophilia (Table 1). However, healthcare financing in Chile remains inefficient and inequitable [17, 18], with marked differences in access and health outcomes between the private and public systems [19].

**Table 1:** Programs available to patients with Rare Diseases in Chile

Name	Disease(s) or tests	Target Population	Coverage
Explicit Guarantees in Health GES (Law N° 19,966) [22]	• Hemophilia • Cystic Fibrosis	Individuals with suspected or confirmed diagnosis	Diagnostic confirmation (excludes molecular diagnosis and genetic counseling), follow up and therapies
Coverage for High Cost medications and devices (Law 20.850 “Ley Ricarte Soto”) [20]	• Mucopolysaccharidoses types I, II, VI • Tyrosinemia Type I • Gaucher Disease • Fabry Disease • Hereditary Angioedema • Pancreatic neuroendocrine tumors • Generalized dystonia • Multiple sclerosis • Primary immunodeficiency • Huntington disease • Epidermolysis bullosa • Amyotrophic lateral sclerosis • Bilateral severe-to-profound sensorineural hearing loss • Unresectable or metastatic gastrointestinal stromal tumors	Individuals with suspected or confirmed diagnosis	Specific high cost medications
Newborn screening Program [23]	• Phenylketonuria	All newborns	Screening, confirmation, follow up, dietary interventions
Expanded newborn screening pilot [24]	• Cystic fibrosis • Other inborn errors of metabolism	All newborns	Under evaluation

An additional financial protection system for high-cost diagnoses and treatments was recently established, Law 20.850, or Ricarte Soto Law (RSL), which, through general taxes, funds medications, medical devices or nutrition for 29 health conditions including 14 RDs like Gaucher and Fabry disease, multiple sclerosis, epidermolysis bullosa, amyotrophic lateral sclerosis and Huntington disease (Table 1) [20]. Patients with other RDs do not have guaranteed access to diagnosis or treatments [21].

### Clinical services and access to diagnosis and therapy

There are 33 practicing clinical geneticists in Chile [25]. This is a third less than the recommended ratio of 0.75 full-time equivalents per 250,000 inhabitants [26]. Since the country lacks formal professional genetic counseling training program, this intervention is primarily provided by clinical geneticists. There are no genetic counseling training programs in Chile, and there is only one professional genetic counselor trained abroad, who focuses mainly on hereditary cancer risk assessment and counseling [27]. This evidences the lack and necessity of these healthcare professionals who play an important role in addressing psychosocial dimensions of RD diagnoses [28]. Chile also faces a shortage of trained and certified laboratory geneticists. Indeed, genetic counseling and laboratory genetics are not recognized as specialties in the Chilean healthcare system.

Regarding molecular diagnosis, over 15 clinical cytogenetic laboratories offer karyotype and fluorescence in situ hybridization (FISH) analysis and only 3 laboratories offer molecular genetic tests such as multiplex ligation-dependent probe amplification (MLPA), microarrays and targeted Sanger sequencing for a limited number of genetic diseases. FONASA partially covers these tests for some genetic RDs (Table 2) [29] but they have a low diagnostic rate for RDs, leaving patients with complex genetic conditions without coverage for adequate testing and consequently without a diagnosis that can inform therapeutic decisions. Even some covered diseases (Table 1) lack of a proper diagnostic approach to molecular testing and genetic counseling. Only one clinical next-generation sequencing (NGS) panel for Rasopathies is available locally, but none for other RDs or complex undiagnosed genetic conditions. Therefore, more comprehensive NGS based genetic tests (e.g. whole-exome sequencing (WES)) are typically sent to international clinical laboratories and paid out-of-pocket by families who can afford them, generating substantial inequalities in access.

**Table 2:** Clinical services available to patients with Rare Diseases in Chile

Name	Disease(s) or tests	Target Population	Coverage
Laboratory diagnostic confirmation (FONASA): Karyotype and single probe FISH [29]	• Congenital anomalies • Cognitive disabilities • Recurrent miscarriage and others	Individuals with suspected chromosomal abnormalities	Testing price, according to public or private insurance.
Laboratory diagnostic confirmation (FONASA): MLPA [29]	• 22q11 microdeletion syndrome • Williams syndrome • Charcot-Marie-Tooth type 1A • Simpson-Golabi-Behmel syndrome • Coffin- Siris syndrome • X-linked ichthyosis • Other recurrent microdeletion and microduplication syndromes	Individuals with suspected sub microscopic chromosomal abnormalities	Testing price, according to public or private insurance.
Laboratory diagnostic confirmation (FONASA): MS-MLPA [29]	• Prader–Willi syndrome • Angelman syndrome • Silver-Russell syndrome • Beckwith-Wiedemann syndrome	Individuals with suspected chromosomal methylation abnormalities	Testing price, according to public or private insurance.
Laboratory diagnostic confirmation (FONASA): Sanger sequencing [29]	• Up to 5 amplicons for molecular confirmation of pathogenic/likely pathogenic single nucleotide variants in known genes	Individuals with suspected monogenic conditions due to pathogenic or likely pathogenic variants in known genes	Testing price, according to public or private insurance.

Finally, the reduced availability and extremely high prices of orphan drugs constitute another barrier for therapies, and patients are bringing their cases of lack of coverage from insurers to court, which has led to an increasing trend in the judicialization of healthcare services [30].

### Economic prioritization criteria for RDs

In Chile, both GES and RSL consider several criteria in their prioritization processes to decide which health conditions and interventions each program will cover, according to criteria established by law [20, 22]. GES broadly includes the burden of disease, budget impact, social preferences, and cost-effectiveness evaluations, while RSL uses the evidence-to-decision methodology, which considers the cost of diagnoses or treatments (must exceed a threshold defined by law) and the rating evidence of interventions using the Grading of Recommendations Assessment, Development, and Evaluation (GRADE) approach [31]. The latter may entail drawbacks for RDs, often lacking high-quality evidence and might also affect the cost-effectiveness analyses of treatments for these disorders [32]. Furthermore, the high prices of these treatments impact the cost of such analyses [9, 33–35], which justifies the need for a higher willingness to pay for RD interventions [34]. Unfortunately, traditional economic evaluation methods do not necessarily capture a complete notion of the social value and societal preferences, leaving RD patients without coverage and proper healthcare. All these issues regarding prioritization criteria, added to the minimal amount of resources allocated to LRS constitute significant downside for access to diagnosis and treatments, especially when considering the substantial economic impact they pose for patients, families, communities, the healthcare system, and society [9].

### Regulation

Other Latin-American countries, such as Argentina, Colombia, and Peru have specific laws for RDs that recognize them as having a special status or be national interest or that guarantee access to treatment or social protection (Fig. 1) [2]. In Chile, although the legislators have recognized a minimum level of access to the essential material components of the constitutional right to health through the GES law [36], RDs were not considered. Therefore, the coverage of RDs was later complemented with another national program oriented toward high-cost drugs through the RSL, which is not specifically focused on RDs either [37]. RDs were left without their own legal structure to guarantee financial coverage and must compete with other common high-cost diseases that more easily satisfy the prioritization criteria mentioned earlier.

### Patient groups

Patients and patient advocacy groups play an important role in the establishment and adoption of healthcare policies for RDs and orphan drugs. Patient groups can provide prevalence data, estimates on the uptake of treatments, and valuable insights into what they consider to be important regarding quality of life [38]. They can also indicate barriers in drug access and treatments for both patients and caregivers, which are crucial for studies on the proper use of healthcare resources [39]. Associations from Latin-American countries have been involved in awareness campaigns, providing legal advice, and informing patients about referral centers if the country has them (for example, the Federation of Uncommon Diseases Argentina). Also, Latin America RDs associations lead the formation of work teams with scientists, clinicians and politicians for law development, such as the screening of rare metabolic diseases in newborns (Federation of Rare Diseases Colombia). In Chile, the Federation for Rare Diseases (FECHER) and the Federation of Uncommon Diseases Chile (FENPOF) are the main patient advocacy groups, composed of patients and their relatives/caregivers, healthcare professionals, and citizens who understand these issues. FECHER actively contributed to the development of the RSL and currently aims to be the official channel for all patients, supporting and defending their rights in health committees where regulations or bills related to RDs are discussed. FENPOF is an emerging organization addressing collaborative work with leaders of RD groups seeking treatment accessibility and legislators. Both organizations aim at increasing awareness and communication about RD to the general public and policymakers in the Ministry of Health and Congress for the development of RD regulations. They also establish alliances with Chilean healthcare providers, scientific research centers, and universities to promote RD research.

### Research

In Chile, biomedical research occurs almost exclusively in universities and is funded primarily by competitive peer-reviewed grants awarded by the National Research and Technology Funding Program (CONICYT), according to the scientific merit of each proposal. Investigator-driven collaborations with foreign research groups also play an important role. Nevertheless, neither special support nor national or private initiatives have arisen to promote research or clinical trials on specific RDs, and RD projects must compete with other, more prevalent conditions that are often more recognized by the community. In a retrospective analysis of CONICYT funds granted from 1999 to 2017, only 37 of them focused on different aspects of RDs, out of 11,588; that is only 0.32% of government-funded projects. Also, only few research groups are actively working in certain rare diseases in Chile (e.g., Epidermolysis bullosa, lysosomal storage diseases, 22q11.2 microdeletion syndromes [40–42]). Two other research groups, namely Chile Genómico [43] and 1000 Genomas Chile [44], are focused on ancestry and genomic variation. Regarding clinical trials, the current state of RSL contains specific provisions intended to protect the enrolled patients against any unwanted effects or unforeseen accidents. RSL establishes civil liability insurance and the presumption that any harm to the health of a patient participating in a clinical trial occurred due to the research, which has significantly increased costs and barriers to conducting a trial in Chile [45]. All of the above indicates that the limited research in RDs, sustained by the efforts of a few research groups, has not been supported by a scientific policy that prioritizes research with societal impact. The newly established Ministry of Science, Technology, Knowledge, and Innovation [46], one of whose functions is to coordinate the stakeholders of the national science system, should play a key role when discussing public policies for RDs.

## Charting the way forward

The World Health Organization (WHO) declared health care a basic human right in 1948. Along that line, Chile is moving toward Universal Health Coverage (UHC) through initiatives such as the recent creation of a National Cancer Program [47]. More efforts should be made to improve equitable access to healthcare services for underrepresented and vulnerable populations like RD patients, especially considering the country’s commitment to sustainable development goals of UHC, “leaving no one behind” (SDG3.8) [48]. In this sense, the lack of financial risk protection for RDs hinders the attainment of UHC in Chile. The lack of resources for clinical services and financial coverage, and the current legislation related to RDs in Chile pose regulatory challenges that could be addressed in a special law for the care of patients with RDs. This law should be complemented by regulatory changes in other related areas, such as clinical research, the use of personal health information, telemedicine, data protection, and genetic tests, among others.

This special law should be grounded on ethical principles such as justice as fairness. This approach is an adequate framework to ethically support why societies/countries should establish public policies for RDs to protect the most disadvantaged in society [49]. For priority setting, the Accountability for Reasonableness (AFR) model establishes basic criteria for fair resource allocation, such as (i) publicity (transparency including reasons), (ii) relevant reasons (as judged by appropriate stakeholders), (iii) revisability (when new evidence or arguments), and (iv) enforceability (assurance that other conditions are met) [50]. However, this is a minimum ethical requirement and does not guarantee consensus in decisions about the financing of high-cost diagnoses and treatments. This is why, to address practical moral problems, it is necessary to integrate principles that are closer to the collaborative and updated discussion carried out by international organizations such as the International Rare Diseases Research Consortium (IRDiRC) [51] that promotes an ambitious vision of enabling all people living with a rare disease to receive an accurate diagnosis, care, and available therapy within one year of coming to medical attention.

We propose an interdisciplinary approach to enable the design and implementation of public policies from an integral social perspective, covering all epidemiological, economic, ethical, and regulatory analyses. This approach, together with the design of a national regulatory proposal in this area should develop methodological guidelines to evaluate current and future policies related to RDs, including ethical guidelines for the use of biological samples and genetic data, and creation of research biobanks. Instead of considering each RD as a single condition, which reduces its epidemiological impact on public health policies, a robust policy should consider RDs together, grouping each known RD and the rare undiagnosed conditions into a single disease category. This grouping would shed light on the magnitude of the problem, facilitating its inclusion in the conversation about health coverage. We list a set of six proposals that we believe will help close the aforementioned gaps in equitable access.

- **Proposal 1.** To establish a national centralized registry of RD patients to obtain specific local as well as integral grouped national evidence, including epidemiology, but also social and economic impact and evaluation of the quality of life of patients and family/caregivers, and preferences regarding patient’s private health information, including genetic data. This registry should be dynamic and keep track of any diagnostic change if new evidence arises. Recently, a national registry for congenital anomalies (RENACH) was established [52]. A similar model can be implemented, with input from clinicians as well as from patients and families.
- **Proposal 2.** To elicit social preferences related to healthcare and financial coverage of RDs using qualitative methods with the general population, patient support groups, scientific societies, decision-makers, and other stakeholders. This information would provide a basis to adjust the existing prioritization algorithm for coverage in the National Health Care system for RD specific needs, taking into consideration the public opinion in the setting of national healthcare priorities [53, 54].
- **Proposal 3.** To improve access to clinical genetic services by increasing the number of providers in clinical/medical genetics, recognizing the specialty of clinical laboratory genetics, promoting training on RDs among primary care health teams to improve diagnostic suspicion and creating programs in genetic counseling for nurses, midwives, and other certified health professionals. To adequately serve the geographically dispersed population, a national interconnected network of clinical services for RDs should be established that can take advantage of existing public Telemedicine. A free public information portal of RD should be established supported by the Ministry of Health to improve knowledge among people.
- **Proposal 4.** To improve the performance of the healthcare system in the procurement of drugs and medical devices for RDs. It is essential to encourage regional cooperation in price negotiation, increase purchasing power, and enhance competition among pharmaceutical sellers. An example of these types of arrangements is the WHO/PAHO Strategic Fund, a mechanism to facilitate the acquisition of strategic health supplies for the Latin America region [55]. In this context, efforts could be made to include drugs associated with RDs in the Strategic Fund list of products and to purchase them through this mechanism.
- **Proposal 5.** To promote and develop local and international research partnerships for scientific and clinical collaboration. An obvious opportunity exists for synergy between research groups focused on RDs and normal human genetic variation to generate an integrated Chilean database of genomic variants. Work is progressing to move towards that goal. The newly established Ministry of Science, Technology, Knowledge, and Innovation [46] is expected to promote the growth and development of sciences and funding; we propose the establishment of specific programs for RDs. Regarding clinical trials, the legislators are considering a modification of the RSL, to promote the development of clinical trials in Chile, reducing the barriers but protecting the patients. All relevant stakeholders, clinical, scientific, political, and patients, should take part in this conversation.
- **Proposal 6.** To promote and facilitate the creation of a Latin-American cooperation network, to encourage the development, establishment, and dissemination of knowledge and new mechanisms for the diagnosis, treatment, and follow-up of RD patients, as well as the implementation of networks of sample and data banks at a regional level, similar to EURORDIS-Rare Diseases-Europe [56].

## Conclusions

It is necessary to discuss and implement a regulatory strategy for RDs in Chile. A comprehensive health policy must consider not only the financing of high-cost diagnoses and treatments but also develop a set of tools that provide sustainability for a comprehensive strategy. Epidemiology in RDs is of vital importance as a reference point to formulate public policies. Determining the magnitude of the problem in Chile through a comprehensive national registry of RD patients, instead of relying on epidemiological estimates from the literature, should be the starting point. Also, considering public participation in setting priorities for public spending on healthcare would turn decision-making by the authority more inclusive and participatory, relevant for healthcare systems with budgetary restrictions.

As recently recommended by the OECD [57], Chile should strengthen its public health policy through genomic medicine, and increase “genetic literacy” among health professionals and the public. Investing in genomic education of healthcare professionals and biomedical scientists should be prioritized over, for example, the acquisition of expensive genomic equipment [58, 59]. In this line, the promotion of international strategic partnerships for scientific research and clinical collaboration is a key factor for resource-limited research settings. Collaborations with international well-funded research groups create benefits for all parties involved, through training opportunities, knowledge transfer, and participation in multicenter projects to study unique cases and patients with rare clinical features, with different genetic backgrounds [60]. The Undiagnosed Diseases Network (UDN) [61] in the U.S. and IRDiRC [51] are good examples of successfully collaborative research initiatives, and Chile should seek to actively participate in these programs. Patient organizations play an important role in this regard, elevating the debate relating to equity and social protection and intervening on public policies projects and research. Patient organizations should strengthen the cohesiveness and strategies of joint work, locally and regionally through a Latin American network to raise awareness, support education, research programs and drug/treatment development. Patient-driven initiatives like EURORDIS are good references.

Finally, the recent Chilean commitment to the Asia-Pacific Economic Cooperation (APEC) Action Plan on Rare Diseases [62], in line with the present proposals, is a step forward toward UHC for RDs. Such a policy would dramatically benefit an important group of people, RD patients and families, who have been historically postponed and unprotected.

## Data Availability

Not applicable.

## Abbreviations

APEC: Asia-Pacific Economic Cooperation
CONICYT: National Research and Technology Funding Program
EU: European Union
FECHER: Chilean Federation for Rare Diseases
FENPOF: Federation of Uncommon Diseases Chile
FISH: Fluorescence in situ hybridization
FONASA: Chilean National Health Fund
GES: Explicit Guarantees in Health
GRADE: Grading of Recommendations Assessment, Development, and Evaluation
IRDiRC: International Rare Diseases Research Consortium
ISAPRE: Chilean private health insurers
MLPA: Multiplex ligation-dependent probe amplification
NGS: Next-generation sequencing
RDs: Rare diseases
RENACH: National Registry for Congenital Anomalies
RSL: Law 20.850, or Ricarte Soto Law
UDN: Undiagnosed Disease Network
UHC: Universal Health Coverage
WES: Whole-exome sequencing
WHO: World Health Organization
